# The role of Magnetic Resonance Imaging and Visual Evoked Potential in management of optic neuritis

**DOI:** 10.11604/pamj.2014.17.54.2462

**Published:** 2014-01-25

**Authors:** Suha Mikail Al-Eajailat, Mousa Victor Al-Madani Senior

**Affiliations:** 1 Senior ophthalmology specialist at King Hussein Medical Center, Neuro-ophthalmology fellowship, Moorfield's Eye Hospital, London, UK; 2 Ophthalmology specialist at King Hussein Medical Center, Neuro-ophthalmology fellowship, Addenbrooke's Hospital, Cambridge, UK

**Keywords:** Optic neuritis, steroids, recurrence, visual evoked potential

## Abstract

**Introduction:**

To report our experience in management of patients with optic neuritis. The effects of brain magnetic resonance imaging and visual evoked potential on management were investigated

**Methods:**

This is a four years clinical trial that included patients presenting with first attack of optic neuritis older than 16 years with visual acuity of less than 6/60 and presentation within first week of illness. Brain magnetic resonance imaging and visual evoked potentials were done for all patients. Patients were classified into three groups. First group received placebo, second received oral steroids and third received intravenous and oral steroids. Primary outcome measure was improvement in visual acuity.

**Results:**

A total number of 150 patients were enrolled in the study. Ocular pain was seen 127 patients Relative afferent pupillary defect in 142 patients and color vision impairment in 131 patients. Abnormal MRI findings were seen in 84 patients. Pattern reversal VEP was abnormal in all patients. Using oral or intravenous steroid resulted in faster recovery but did not affect the final visual outcome. Recurrence rate was higher in patients with multiple MRI lesions and diminished VEP amplitude. Using intravenous steroids decreased recurrence rate in patients with three and more MRI lesions and non recordable VEP response.

**Conclusion:**

MRI and pattern reversal VEP are recommended to be done in all patients presenting with optic neuritis. We advise to give intravenous methyl prednisolone in patients with multiple MRI white matter lesions and non recordable VEP at presentation.

## Introduction

Optic neuritis is an inflammation of the optic nerve. It could be idiopathic or associated with demyelinating, autoimmune, infectious and inflammatory conditions [1, 2]. Acute demyelinating optic neuritis associated with multiple sclerosis is the most common cause of optic neuritis [3–5]. Patients with optic neuritis usually present with sudden visual loss which is usually rapid. The severity of visual loss ranges from mild to no light perception. Some patients may present with normal vision when their central vision is preserved and the peripheral vision is affected [6]. Others may present with dyschromatopsia due to color vision involvement [7].

The majority of patients with acute demyelinating optic neuritis recover their vision. The peak of visual loss usually occurs two weeks after the disease onset. Recovery starts after three weeks and the majority shows complete recovery after five weeks [8–10]. Less than 10% of patients were reported to suffer from visual impairment one year after illness and 35% of patients may develop recurrent attacks [8, 11]. Many studies investigated how to prevent visual impairment and recurrence rate. The Optic Neuritis Treatment Trial (ONTT) is the pioneer of such studies. It is a multicenter controlled clinical trial funded by the National Eye Institute of the National Institutes of Health in the United States. Although the primary objective of the trial was to assess the efficacy of corticosteroids in the treatment of optic neuritis, the trial also provided invaluable information about the clinical profile of optic neuritis, its natural history, and its relationship to multiple sclerosis [11]. ONTT results have shown that the use of intravenous steroids results in faster visual recovery but does not affect the final visual outcome and that the use of oral steroids alone increased the risk of recurrence. Although many studies supported such results [12–14], there are studies that found the opposite [15]. In our opinion, there are two major drawbacks about ONTT that contributes to controversy. The first one is that ONTT is an old study conducted in 1988 and the second is the relative small sample enrolled (455 patients).

In our study, we selected patients with visual acuity of less than 6/60 as many physicians agree not to treat patient with mild to moderate visual loss. The aim of the study was to report our experience in management of cases presenting with optic neuritis. We investigated whether MRI and VEP abnormalities can affect management protocol.

## Methods

This is a four years clinical trial. Patients with optic neuritis attending neuro-ophthalmology clinic during the period between January 2008 and January 2012 were enrolled in the study and were evaluated by senior neuro-ophthalmology specialist. Inclusion criteria included patients older than 16 years, visual acuity of less than 6/60 at presentation, no previous attacks of optic neuritis and presentation within first week of illness. Data collected included clinical presentation, course, management and outcome of illness. Ocular examination included best corrected visual acuity by Snellen's E-chart, anterior segment examination by slit lamp, posterior segment examination after mydriasis with + 78 lens, optic nerve function tests including ishihara pseudoisochromatic color plates for color vision and testing for relative afferent pupillary defect, and Humphrey visual field assessment using 30-2 program. Color vision was considered impaired when patient missed six or more plates out of seventeen plates. T2 weighted brain magnetic resonance imaging (MRI) and pattern reversal visual evoked potential (VEP) using International society for Clinical Electrophysiology of Vision (ISCEV) recommendations were done for all patients. Significant white matter MRI lesion was considered when measured more than 3 mm in diameter. Pattern reversal VEP amplitude and latency were measured for large and small check patterns.

Patients were classified into three groups (fifty patients each). First group received placebo treatment, second group received 1mg/kg/day oral prednisolone for two weeks and third group received intravenous 1 gram methyl prednisolone sodium succinate daily for 3 days followed by 1mg/kg/day oral prednisolone for 11 days with rapid tapering (20 mg on day 12, 10 mg on day 13, nothing on day 14 and 10 mg on day 15). Patients were followed up weekly for one month, monthly for three months and every three months for 2 years. Assessment of visual acuity and optic nerve function tests were done in each visit. Primary outcome measure was improvement in visual acuity. Secondary measures included color vision and visual field improvement. P-value was used for statistical analysis and was considered significant when P < 0.05.

## Results

A total number of 150 patients were enrolled in the study. Age range was 16.4 years to 52.9 years (mean 32.7 years). Female outnumbered males (2.1 to 1 ratio). Ocular pain was seen 127 patients (84.7%). Relative afferent pupillary defect in 142 patients (94.7%) and color vision impairment in 131 patients (87.3%). Visual field defects were seen in all patients with central and paracentral being the most common (95, 63.3%). Other visual field defects were arcuate, altitudinal, nasal step, hemi field and non specific changes (Table 1). Abnormal MRI findings were seen in 84 patients (56%). One lesion was seen in 41 patients (27.3%), two lesions in 16 patients (10.7%), three lesions in 15 patients (10%) and four and more lesions in 12 patients (8.0%). Pattern reversal VEP was abnormal for large and small patterns in all patients. P100 latency was prolonged in 135 patients (90%). Fifteen patients (10%) had non recordable VEP, and 42 patients (28%) had reduced P100 amplitude (Table 2).

**Table 1:** Clinical picture of patients

Feature		Number	Percentage
Ocular pain		127	84.7%
Relative afferent pupillary defect		142	94.7%
Color vision impairment		131	87.3%
**Visual field defects**	central and paracentral	95	63.3%
	arcuate	15	10%
	altitudinal	8	5.3%
	nasal step	6	4.0%
	hemi field	4	2.7%
	non specific	22	14.7%
	Total	150	100%

**Table 2:** MRI and visual evoked potential findings

Feature		Number of patients	Percentage
**Abnormal MRI**	one lesion	4184	27.3%
	two lesions	16	10.7%
	three lesions	15	10%
	four lesions and more	12	8%
	Total	84	56%
**Abnormal VEP**	prolonged latency	135	90%
	non recordable	15	10%
	reduced amplitude	42	28%
	Total	150	100%

Regarding patients who received placebo treatment, forty patients improved after 3 weeks and 43 patients after 4 weeks of onset. Five patients had visual acuity of less than 6/12 after one year. Patients receiving oral treatment alone, 43 patients improved after 3 weeks, 47 patients after 4 weeks and 4 patients had visual acuity of less than 6/12 after one year. In patients with intravenous steroids the numbers of patients were 42, 47 and 4 respectively. Recurrence or second eye involvement during a two years period occurred in five patients with placebo, five patients with oral and one patient with intravenous steroids (Table 3).

**Table 3:** Course of illness in patients treated with placebo, oral or intravenous steroids

Feature	Placebo	Oral	Intravenous	p-value
Improvement after 3 weeks	40	43	42	> 0.05
Improvement after 4 weeks	43	47	47	> 0.05
Visual acuity of less than 6/12 after 1 year	5	4	4	> 0.05
Visual acuity of less than 6/12 after 2 year	4	3	4	> 0.05
Visual acuity of less than 6/60 after 2 years	1	1	1	> 0.05
6/6 visual acuity	35	37	35	> 0.05
Recurrence within 2 years	5	5	1	< 0.05

Recurrence within 2 years occurred in 11 patients; six of them had four or more brain MRI and ten of them had three or more lesions. Non recordable VEP was seen in 10 of them. We divided patients according to number of brain MRI lesions and VEP results evenly in each group. The total number of patients with non recordable VEP was 15. Each treatment group had 5 of them. In addition, each treatment group had 5 patients with three brain MRI lesions and 4 patients of four and more lesions. The use of intravenous steroids significantly reduced the incidence of recurrence only in patients with three and more brain MRI lesions or non recordable VEP response (Table 4, Table 5).

**Table 4:** Recurrence according to MRI findings and treatment modality

Treatment modality		Number of patients	Recurrence	p-value
**Placebo**	no lesion	21	0	> 0.05
	one lesion	14	0	> 0.05
	two lesions	6	0	> 0.05
	three lesions	5	2	< 0.05
	four and more	4	3	< 0.05
	Total	50	5	< 0.05
**Oral**	no lesion	22	0	> 0.05
	one lesion	14	0	> 0.05
	two lesions	5	1	> 0.05
	three lesions	5	2	< 0.05
	four and more	4	2	< 0.05
	Total	50	5	< 0.05
**Intravenous**	no lesion	23	0	> 0.05
	one lesion	13	0	> 0.05
	two lesions	5	0	> 0.05
	three lesions	5	0	< 0.05
	four and more	4	1	< 0.05
	Total	50	1	< 0.05

**Table 5:** Recurrence according to VEP findings and treatment modality

VEP finding		Over all	Recurrence	p-value
**Placebo**	non recordable	5	4	< 0.05
	other	45	1	> 0.05
	Total	50	5	< 0.05
**Oral**	non recordable	5	5	< 0.05
	other	45	0	> 0.05
	Total	50	5	< 0.05
**Intravenous**	non recordable	5	1	< 0.05
	other	45	0	> 0.05
	Total	50	1	< 0.05

## Discussion

We selected patients with visual acuity of less than 6/60 in our study. Therefore, all of our patients have decreased vision. Visual acuity at presentation ranged from 6/120 to 6/60 in 117 patients (78%). Seven patients (4.7%) had hand movement vision, three (2%) with light perception and two (1.3%) with no light perception. Twenty one patients (14%) had visual acuity between counting fingers one meter to three meters. Visual acuity was the primary outcome measure we used. Improvement was considered when patient visual acuity improved by more than 50%, i.e. from 6/60 to 6/30 or from CF 3 m to 6/60. Ocular pain occurred in 127 patients (84.7%). The pain proceeded visual impairment in 99 patients and occurred after visual impairment in 28 patients. It resolved within the first week of illness in the majority of patients. Relative afferent pupillary defect (RAPD) was seen in 142 patients. RAPD occurs in all patients with optic neuritis. The remaining 8 patients, RAPD was not evident because contra lateral optic nerve was affected by other pathology. Color vision impairment was seen in 131 patients. We used Ishihara color plates and abnormality was considered when 6 or more plates were missed. The accuracy of Farnsworth-Munsell 100-Hue test is better than Ishihara plates for detection of various optic neuropathies [7]. In the ONTT, Ishihara color plates were abnormal in the affected eye in 88%, whereas the Farnsworth-Munsell 100-Hue test was abnormal in 94% [11].

Visual field defects occurred in all our patients. We used Humphrey 30-2 program. The most common visual field defect was central and paracentral scotoma. Other defects were arcuate, altitudinal, nasal step, hemi field loss and non specific visual field changes. Twelve patients had visual acuity of hand movement or less and the visual field could not be done for them and was labeled as non specific. Table 1 shows the clinical presentation of patients. Magnetic resonance imaging is important diagnostic work up in patients presenting with optic neuritis. The presence of two or more white matter lesions (3 mm or larger in diameter, at least one lesion periventricular or ovoid) suggests high risk for multiple sclerosis [11, 12, 14, 16]. Also, it helps to rule out other pathology when the diagnosis is doubtful. In our series, the number of patients with no MRI pathology was 66 (44%). Forty one patients had one lesion, sixteen had two lesions, fifteen had three lesions and twelve had four and more lesions (Table 2). We tried to distribute patients with same number of MRI lesions evenly in each treatment group in order to see whether the management of patients is affected by the presence of MRI lesions.

Pattern reversal visual evoked potential latency is prolonged in all cases of demyelinating optic neuritis. VEP can be helpful to diagnose cases of subclinical and chronic optic neuritis. The amplitude of VEP may be normal or reduced. If the attack is severe enough it may give us non recordable VEP. Fifteen of our patients had non recordable VEP. We also distributed these patients evenly in treatment groups (5 patients each) in order to see whether the management of patients is affected by VEP results.

Forty patients of placebo group (80%) showed improvement after 3 weeks and 86% showed improvement after 4 weeks. Among patients enrolled in the ONTT who received placebo, visual acuity began to improve within 3 weeks of onset in 79% and within 5 weeks in 93% [11]. 86% and 94% of our patients who received oral treatment showed improvement three and four weeks respectively while the figures were 84% and 94% in patients with intravenous group (Table 3). Although this is considered as a faster recovery when compared with placebo group; however, this was not statistically significant. The visual outcome one year after illness did not show significant difference between the three groups. Visual acuity of less than 6/12 was seen in 10%, 8% and 8% of placebo, oral and intravenous groups respectively. After two years, visual acuity of less than 6/12 was seen in 8%, 6% and 8% of patients respectively and visual acuity of less than 6/60 in 2 years in 2% of each group. Visual acuity of 6/6 was seen in 70% of placebo and intravenous group and 74% of oral group. All of these figures were statistically not significant.

The recurrence rate of optic neuritis in our study within two years was 10% for patients with placebo and oral treatment and 2% for patients who received intravenous steroids. This was statistically significant and supports the results of other studies. When we classified patients further based on MRI and VEP findings, we obtained different results. Table 4 shows the effect of treatment based on MRI lesions. When there was one lesion or less, there was no recurrence whether patients treated with placebo, oral or intravenous steroids. In patients with two lesions only one patient (2%) of oral group showed recurrence; however, this was not statistically significant. Recurrence rate was higher in patients with three and four or more lesions (4% and 6% in placebo and 4% and 4% in oral compared to zero and 2% in intravenous group). This was statistically significant and the conclusion made was that the use of intravenous steroids in patients with three or more MRI lesions can prevent recurrent attacks of optic neuritis. Regarding the effect of VEP on treatment, we found similar results (Table 5). In patients with recordable VEP, recurrence rate was 2% in patients with placebo and zero in patients with oral or intravenous steroids. When VEP was non recordable, recurrence was 8%, 10% and 2% in three groups respectively. Again the conclusion made was that the use of intravenous steroids in patients with non recordable VEP can prevent recurrent attacks of optic neuritis.

We reviewed the literature for studies with similar results. There are studies that showed the importance of MRI on management of optic neuritis [14]. However, we could not find any study investigating the effect of VEP on management of optic neuritis.

## Conclusion

Oral and intravenous steroids have no effect on final visual outcome though they can speed up recovery. The recurrence rate was much less with intravenous steroids only in patients with three or more lesions on MRI or with non recordable VEP. Therefore, brain MRI and pattern reversal VEP is highly recommended to be done in patients with optic neuritis presenting with visual acuity of less than 6/60.
